# In vivo evidence for pre‐symptomatic neuroinflammation in a MAPT mutation carrier

**DOI:** 10.1002/acn3.683

**Published:** 2019-01-02

**Authors:** W. Richard Bevan‐Jones, Thomas E. Cope, P. Simon Jones, Luca Passamonti, Young T. Hong, Tim Fryer, Robert Arnold, Jonathan P. Coles, Franklin I. Aigbirhio, John T. O'Brien, James B. Rowe

**Affiliations:** ^1^ Department of Psychiatry University of Cambridge Cambridge United Kingdom; ^2^ Department of Clinical Neurosciences University of Cambridge Cambridge United Kingdom; ^3^ Wolfson Brain Imaging Centre University of Cambridge Cambridge United Kingdom; ^4^ Division of Anaesthesia University of Cambridge Cambridge United Kingdom; ^5^ Medical Research Council Cognition and Brain Sciences Unit Cambridge United Kingdom

## Abstract

Neuroinflammation occurs in frontotemporal dementia, however its timing relative to protein aggregation and neuronal loss is unknown. Using positron emission tomography and magnetic resonance imaging to quantify these processes in a pre‐symptomatic carrier of the 10 + 16 MAPT mutation, we show microglial activation in frontotemporal regions, despite a lack of protein aggregation or atrophy in these areas. The distribution of microglial activation better discriminated the carrier from controls than did protein aggregation at this pre‐symptomatic disease stage. Our findings suggest an early role for microglial activation in frontotemporal dementia. Longitudinal studies are needed to explore the causality of this pathophysiological association.

## Introduction

Genetic etiologies account for up to a third of cases of frontotemporal dementia (FTD),^1^ and may provide important insights into the pathophysiology of sporadic FTD.^2^, ^3^ While disease arising from genetic abnormalities in microtubule associated protein tau (MAPT) accounts for 5–10% of cases, around 40% of patients with FTD display tau pathology.^1^ Pre‐symptomatic studies of familial FTD have shown cognitive, structural, and network functional connectivity changes preceding symptomatic onset of FTD by several years.^4^, ^5^ The study of pre‐symptomatic mutation carriers with other modalities such as positron emission tomography (PET) may yield insights into early pathophysiological processes.^2^, ^3^ Here, we focus on two key, potentially modifiable processes, neuroinflammation and tau protein aggregation. Neuroinflammation has been implicated in FTD by cerebrospinal fluid,^6^ genetic,^7^, ^8^, ^9^ and PET studies.^10^ Microglial activation may alter brain homeostasis in protective or deleterious manners through inflammatory pathways, cytotoxicity, and changes in neuronal plasticity.^11^ Despite evidence of neuroinflammation in FTD, little is known about their in vivo relationship to protein aggregation and neuronal loss.

Neuroinflammation and tau pathology can be quantified using PET. [^11^C]PK‐11195 is a biomarker of activated microglia and therefore a surrogate measure for neuroinflammation.^12^ [^18^F]AV‐1451 binds preferentially to paired helical filament tau neuropathology 13, 14 but is also sensitive to the straight filament tauopathy present in the 10 + 16 MAPT mutations in familial FTD.^15^ We use both ligands and structural magnetic resonance imaging (MRI) in a pre‐symptomatic carrier of the 10 + 16 MAPT mutation, associated with a straight filament 4 repeat tauopathy, to test the hypothesis that neuroinflammation is an upstream event of tau aggregation and brain atrophy or *vice versa*.

## Methods

A 53‐year‐old female pre‐symptomatic carrier of a 10 + 16 MAPT mutation underwent neuropsychological testing, structural MRI, [^11^C]PK‐11195 PET, and [^18^F]AV‐1451 PET within a 2‐month period. Blood was taken for analysis of C‐reactive protein (CRP), a nonspecific peripheral marker of inflammation, at the time of [^11^C]PK‐11195 PET. A first‐degree relative with the same MAPT mutation had previously been diagnosed with behavioral variant FTD.

Protocols for neuropsychology, MRI, and PET are described in Bevan‐Jones et al.^16^ [^18^F]AV‐1451 PET data were acquired and processed using previously described methods.^17^ Briefly, a simplified reference tissue model (superior cerebellar gray matter reference tissue) was used to calculate the nondisplaceable binding potentials (BP_ND_) in 83 regions of interest (ROI) based on Hammersmith atlas n30r83 modified to include subcortical structures. [^11^C]PK‐11195 PET data were similarly processed into 83 ROIs using the same atlas (reference tissue defined by supervised cluster analysis).^18^ For both tracers, regional BP_ND_ was corrected for CSF partial volume effects. To reduce the PET radiation dose received by healthy volunteers, PET data were compared to separate cohorts of healthy participants of similar age. Fifteen control data sets were obtained for [^11^C]PK‐11195, and 13 for [^18^F]AV‐1451.

For each PET ligand two questions were posed. Firstly, there were areas of the brain with higher BP_ND_ in the MAPT carrier compared to the control group. For each region, a *t*‐score was calculated compared to the control group. Secondly, irrespective of the absolute level of ligand binding, did the distribution of binding across brain regions differ between the MAPT carrier and the control group? For each ligand the parcellated data were converted to individual linear vectors of ROI. These vectors were nonparametrically correlated (Spearman's rho), resulting in a correlation matrix, which was converted to a dissimilarity matrix, and fed into hierarchical cluster analysis based on average linkage distance. The information provided by these ligands was compared by assessing the nonparametric correlation of their distributions in the MAPT carrier and controls separately.

Finally, we compared regional gray matter volumes in the 83 regions which had been calculated from the T1‐weighted MPRAGE images as part of the PET pre‐processing method and parcellated using the same atlas. A general linear model was applied to each region independently to look for differences between the MAPT carrier and [^18^F]AV‐1451 control group including age and total intracranial volume as predictors of no interest.

Data from all three modalities were also compared to an individual with established MAPT behavioral variant FTD, whose full background and [^18^F]AV‐1451 results have previously been published.^15^

## Results

**Figure 1:**
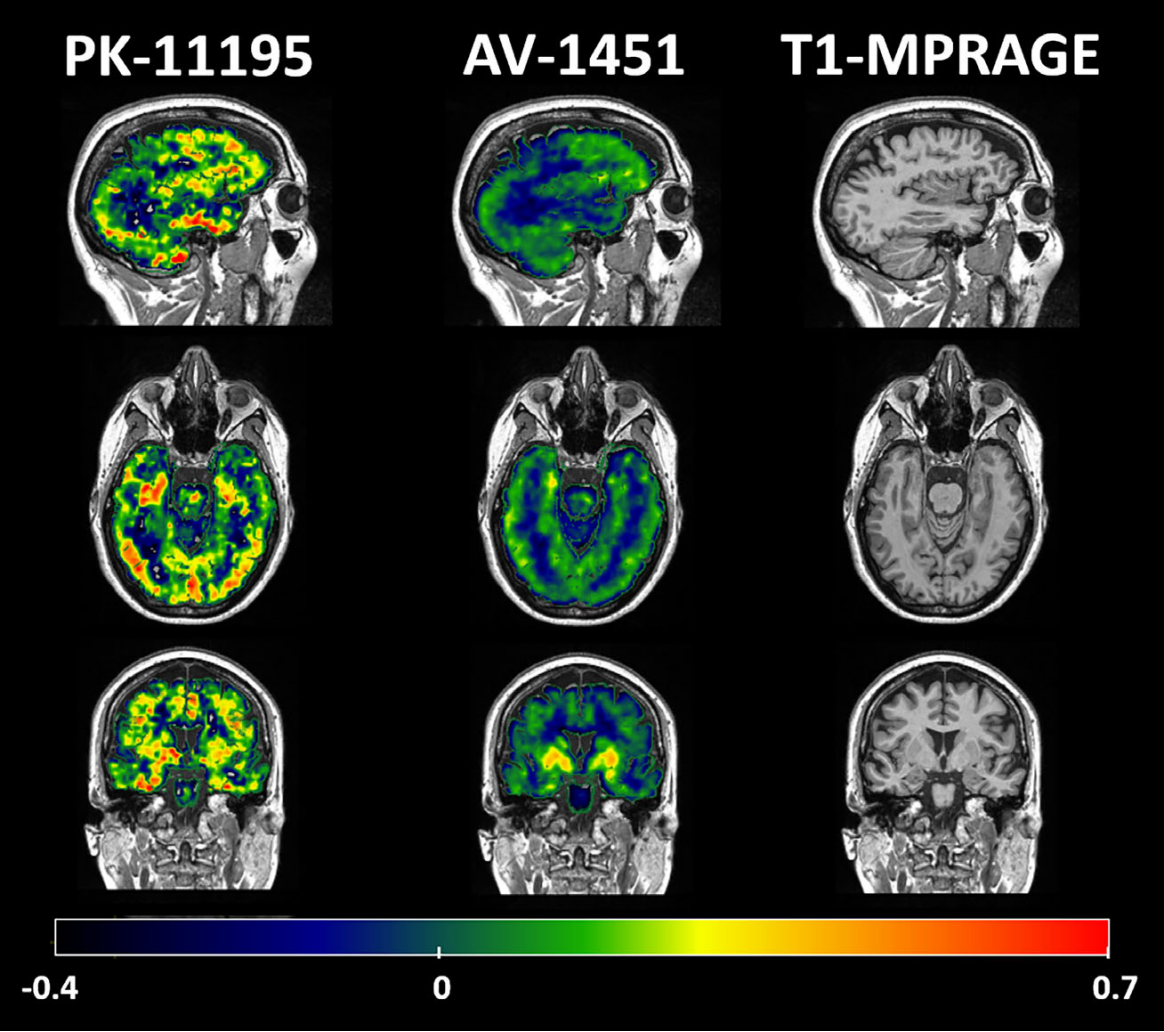
Sagittal, axial, and coronal slices of the raw BP_ND_ maps for PK‐11195, AV‐1495, and T1‐weighted MPRAGE in the MAPT carrier. The BP_ND_ scale bar runs along the bottom of the figure.

**Figure 2:**
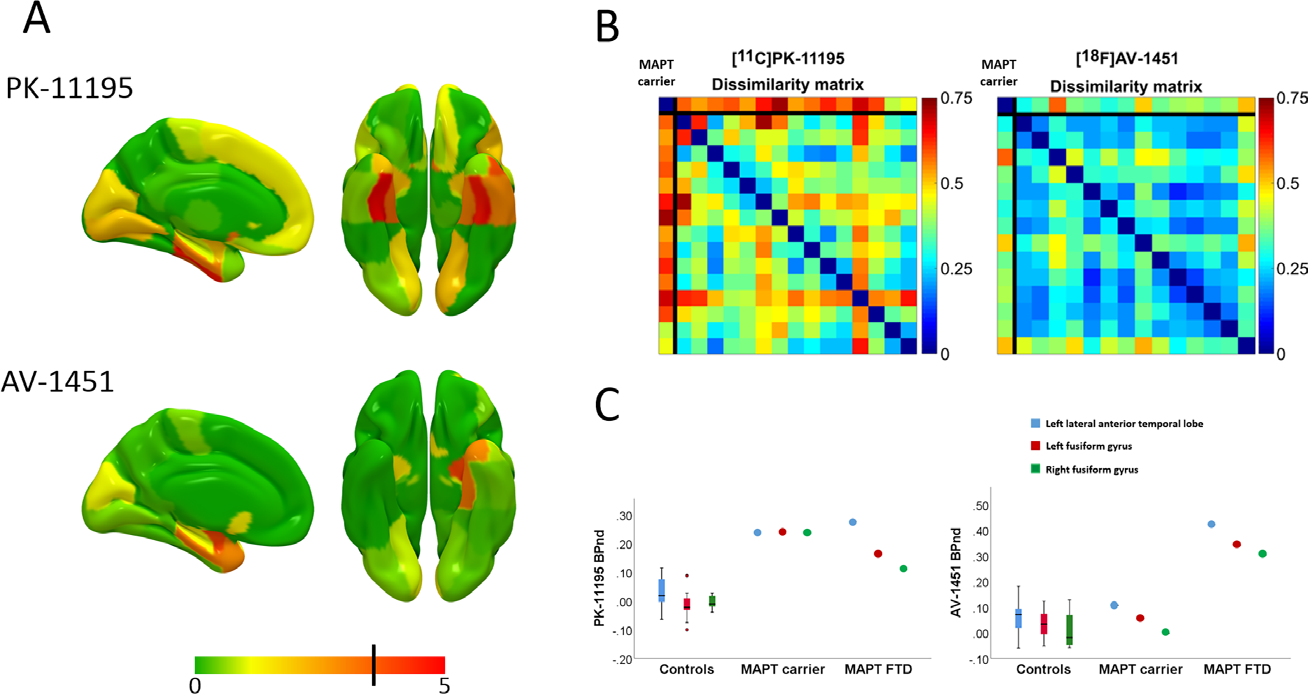
Panel A – un‐thresholded maps of *t*‐scores for the MAPT carrier against controls for PK‐11195 and AV‐1495. Regions colored red (to the right of the black line on the color scale) survived FDR correction within each modality. Panel B – Dissimilarity matrices for across‐individual whole‐brain distributions of PK‐11195 (left) and AV‐1451 (right); for each matrix the case is represented in the first row and column, with each control providing a subsequent row and column. Panel C – PK‐11195 (left) and AV‐1495 (right) binding potential for the MAPT carrier in regions with elevated PK‐11195, in comparison to AV‐1495 binding potential in the control groups and in a more advanced MAPT FTD case previously published by our group.

Demographics and neuropsychological scores for the MAPT carrier and controls are summarized in Table 1. Raw BP_ND_ maps for both [^11^C]PK‐11195 and [^18^F]AV‐1451 alongside T1‐weighted MPRAGE images for the MAPT carrier are displayed in Figure 1. Un‐thresholded *t*‐scores for [^11^C]PK‐11195 and [^18^F]AV‐1451 are shown in Figure 2A for display purposes. The brain regions with elevated [^11^C]PK‐11195 BP_ND_ that survived correction for false discovery rate (FDR) in the MAPT carrier relative to controls were the left lateral anterior temporal lobe (*t*(12) = 3.88, *q* = 0.046), left fusiform gyrus (*t*(12) = 4.70, *q* = 0.014), and right fusiform gyrus (*t*(12) = 10.33, *q* < 0.001). The distribution of [^11^C]PK‐11195 BP_ND_ was clearly dissimilar in the MAPT carrier compared to controls (Fig. 2B). Hierarchical clustering analysis based on average linkage classified the patient radio‐ligand distribution as the most abnormal. Blood sampling at the time of [^11^C]PK‐11195 PET found a CRP <4 mg/L (the lower limit of test sensitivity). No regions showed elevated [^18^F]AV‐1451 BP_ND_ after FDR correction. The distribution of [^18^F]AV‐1451 BP_ND_ was only moderately dissimilar for the MAPT carrier compared to controls (Fig. 1B). Hierarchical clustering analysis based on average linkage classified the patient distribution as the third most abnormal. Atrophy in the right amygdala was the only structural change that survived FDR correction (*t*(10) = 5.26, *q* = 0.031). Figure 1C shows BP_ND_ for [^18^F]AV‐1451 and [^11^C]PK‐11195 in the control group, MAPT carrier and a previously published case with established MAPT behavioral variant FTD.^15^

**Table 1:** Demographic information and neuropsychological test scores for the MAPT mutation carrier and the two control groups

	MAPT carrier	AV‐1451 controls	PK‐11195 controls
Age	53	67 (55–80)	70 (59–84)
Gender (F:M)	Female	7:6	8:7
Education (years)	11	16 (11–19)	14 (10–19)
Addenbrooke's cognitive examination – revised (out of 100)	86	95 (89–99)	93 (79–100)
Frontal assessment battery (out of 18)	16	n/a	n/a
FTD rating scale (%)	80	n/a	n/a

## Discussion

The primary finding is that the pre‐symptomatic phase of MAPT mutation carriage is associated with temporal lobe neuroinflammation in the absence of significant binding of the “tau” PET ligand [^18^F]AV‐1451 (Fig. 2A), even though this ligand has been shown to bind in symptomatic cases with the same mutation.^15^ The multivariate, nonparametric dissimilarity matrices confirmed this by showing that the pre‐symptomatic carrier was clearly discriminated from controls by her [^11^C]PK‐11195 binding distribution, but not her [^18^F]AV‐1451 binding distribution (Fig. 2B). Of note, there was only marginal gray matter atrophy (in a single, small region, the amygdala) in the pre‐symptomatic MAPT mutation carrier. Together, our results suggest limited tau abnormal accumulation and minimal neuronal loss at the time of assessment, despite the presence of neuroinflammation. The relatively small degree and distribution of [^18^F]AV‐1451 binding, indicative of tau burden, also confirms that our proband was in the early phase of a potentially long natural history of neurodegeneration associated with a MAPT mutation.^4^ Overall these findings suggest that microglial activation precedes clinical symptom onset and significant structural changes in this hereditary tauopathy, constituting an early feature rather than a late consequence of neurodegeneration. The hypothesis that microglial activation precedes clinical symptom onset has been previously suggested^19^ and is supported by our comparison with an individual with symptomatic FTD due to the same 10 + 16 MAPT mutation (Fig. 2C). Our pre‐symptomatic carrier demonstrated a similar level of elevation in [^11^C]PK‐11195 binding compared with this symptomatic individual, but did not demonstrate the same elevation of [^18^F]AV‐1451 binding.

There are two obvious potential interpretations of this phenomenon. Firstly, it is possible that microglial activation promotes abnormal tau aggregation. Alternatively, microglial activation might be induced prior to the presence of tau aggregates by oligomeric tau, to which [^18^F]AV‐1451 is less likely to bind. Therefore, while our data suggest an early role for inflammation in MAPT‐related FTD, they do not settle the debate about whether microglial activation promotes tau aggregation, whether it is a reactive or even protective process. This question could be directly assessed in future work by mediation analysis in longitudinally examined cohorts of genetic carriers, such as that afforded by the Genetic Frontotemporal dementia Initiative (GENFI).^4^

This study has the limitations of a single case report, even when compared to a larger control group. However, it illustrates the potential utility of multimodal imaging in pre‐symptomatic stages of FTD‐related diseases to investigate the early pathophysiology of neuropathological subtypes. While our MAPT carrier is pre‐symptomatic, she is approaching the age at which she would be expected to manifest symptoms, and older than the age at which her sibling clinically manifested the disease. She already demonstrates the abnormalities on structural imaging and neuropsychological testing (Table 1) that have been described 5–10 years before symptom onset in genetic FTLD.^4^ However, her disease process is at a relatively early stage and without the clinical features necessary to meet diagnostic criteria for FTD, allowing us to be clear that abnormal microglial activation is not a feature of only late disease. One must also consider the potential for differences in the signal‐to‐noise of the two PET methods and MRI volumetry. Differences in data variance in the two methods would lead to differences in statistical maps, even for equivalent magnitudes of disease effects on inflammation, tau, and atrophy. Against this, however, are the findings from the representational similarity analysis, using measures based on BP_ND_ distribution not absolute binding values. There are also limitations related to [^18^F]AV‐1451. Unlike in Alzheimer's disease, binding in non‐Alzheimer's neurodegeneration is less understood. Concerns regarding specificity relate to the demonstration of “off‐target” binding in the basal ganglia of healthy individuals^14^ and increased binding in FTD cases likely to have TDP‐43 pathology.^20^, ^21^, ^22^ Binding to monoamine oxidase has also been described and is of potential relevance as isoforms may be present in neurons or in reactive astrocytes.^23^, ^24^ However, [^18^F]AV‐1451 is sensitive to the level and distribution of neuropathology in FTD due to the same MAPT mutation as our case.^15^, ^25^ Finally, given the disparity in age between the case and the healthy control group, the potential confound of age warrants discussion. Both [^11^C]PK‐11195 and [^18^F]AV‐1451 binding are potentially influenced by age with previous studies showing increased binding with increasing age in healthy individuals.^26^, ^27^ The presence of increased binding with age would make it more difficult to find differences between a younger case and an older healthy control group rather than *vice versa*, and therefore would not be an explanation of the group differences in [^11^C]PK‐11195 binding found here. This could provide a plausible explanation for the null result found in the [^18^F]AV‐1451 data. However, in the control groups in our dataset no significant effect of age on the binding potential of either ligand at a regional or global level could be found, suggesting that such effects, if present, are likely to be small.

Overall, the ability of PET tracers to detect pathophysiological changes upstream of neuronal loss demonstrates promise for future research in larger cohorts. Using these techniques in pre‐symptomatic mutation carriers may yield insights into the pathophysiology of distinct neuropathological subtypes which, as well as leading to advances in the treatment of genetic forms of FTD, may elucidate the pathogenesis of sporadic FTD and other neurodegenerative tauopathies. Similar studies using larger patient cohorts and longitudinal assessment of the role of neuroinflammation in early‐stage neurodegeneration will improve our understanding of mechanisms of disease with a view to early targeted intervention.

## Conflict of Interest

JOB has acted as a consultant for GE Healthcare and Lilly.
